# Comparison of Prevalence of Metabolic Syndrome Between Korean Emigrants and Host Country Residents in Japan and China-The Korean Emigrant Study

**DOI:** 10.4178/epih/e2010005

**Published:** 2010-05-07

**Authors:** Myung-Hee Shin, Mi Kyung Kim, Zhong Min Li, Hyun-Kyung Oh, Soo Ryang Kim, Miyuki Taniguchi, Jinnv Fang

**Affiliations:** 1 Department of Social & Preventive Medicine, Sungkyunkwan University School of Medicine, Suwon, Korea.; 2 Department of Preventive Medicine, College of Medicine, Hanyang University, Seoul, Korea.; 3 Department of Epidemiology & Statistics, Jilin University, School of Public Health Science, Changchun, China.; 4 Department of Gastroenterology, Kobe Asahi Hospital, Kobe, Japan.; 5 Department of Preventive Medicine, College of Medicine, Yanbian University, Jilin, China.

**Keywords:** Metabolic syndrome X, Obesity, Type 2 diabetes mellitus, Cardiovascular diseases, Korean Emigrant, Epidemiology

## Abstract

**OBJECTIVES:**

This study aims to compare the prevalence of metabolic syndrome between Korean emigrants (KEs) and their host country residents in Japan and China.

**METHODS:**

The Korean Emigrant Study (KES) is a cohort study initiated in 2005 to elucidate the effect of genetic susceptibility and environmental change on hypertension, diabetes, and metabolic syndrome. Equal numbers of KEs and host country residents, aged 30 or over, were recruited from three regions; Kobe-Osaka in Japan (total number=965), Yanbian in China (n=1,019), and Changchun in China (n=949).

**RESULTS:**

The age-adjusted prevalences of metabolic syndrome among KEs in Kobe-Osaka were significantly higher than those among Japanese (in men 24.0% vs. 15.6%, p=0.04, in women 8.4% vs. 2.7%, p=0.01), while the age-adjusted prevalences among KEs in Changchun were similar to those among Chinese (in men 11.7% vs. 16.1%, p=0.37, in women 28.3% vs. 30.1%, p=0.91). The age-adjusted prevalences were generally higher in Yanbian than other regions, and KEs had higher prevalence than Chinese in men but not in women (in men 37.9% vs. 28.3%, p=0.03, women 46.0% vs. 50.6%, p=0.44). The components with significant ethnic differences in prevalence were high blood pressure and abdominal obesity in Japan, and triglyceride in China. The most influential component in diagnosing metabolic syndrome was abdominal obesity in men and triglyceride in women.

**CONCLUSION:**

The prevalence of metabolic syndrome was higher in KEs than in host country residents in Japan but not in China. Abdominal obesity and triglyceride are both discriminating and influential components in metabolic syndrome.

## INTRODUCTION

Metabolic syndrome is a cluster of metabolic disorders characterized by abdominal obesity, hyperglycemia, hyperlipidemia, and hypertension. It is a state of over-accumulation of fat in internal organs and abdominal wall that leads to insulin resistance, and then a rise of the risk of type 2 diabetes mellitus and cardiovascular diseases.

Prevalence of metabolic syndrome, especially among Asians, varies substantially by the type of definition of metabolic syndrome and by the method of selecting study population and measuring metabolic components. The National Cholesterol Education Program Adult Treatment Panel III (NCEP-ATP III) criteria in 2001 defines metabolic syndrome as having any three or more of the following; abdominal obesity (measured in waist circumference, >102 cm for men and >88 cm for women), high serum triglyceride, low high density lipoprotein (HDL)-cholesterol, and high fasting glucose(>=110 mg/dL), and hypertension [1]. In 2005, a revised version of NCEP-ATP III was proposed to incorporate Asian-Pacific specific criteria for waist circumference (>90 cm for men and >80 cm for women) and to lower the criteria for fasting glucose (>=100 mg/dL) [2]. Some studies conducted in Asia defined metabolic syndrome by NCEP-ATP III with Asian-Pacific criteria for central obesity [3-5] or by revised NCEP-ATP III with their own criteria for abdominal obesity (in Japan >=85 cm for men and >=90 cm for women) [6]. In addition, the World Health Organization (WHO) and International Diabetes Federation (IDF) also proposed their own definitions of metabolic syndrome [7, 8]. Therefore, comparison of prevalences requires unification of the definition of metabolic syndrome. Even under the same definition, the prevalence can vary according to the method of population selection. National survey data or a randomly selected study population usually provides the most representative estimate of prevalence, while studies with health check-up participants or an arbitrarily selected population tend to report a wide range of variation. For example, the prevalence of metabolic syndrome among Korean men who participated in health check-ups varies from 9.8% to 34.2% under the same criteria of NCEF-APC III with Asian-Pacific abdominal obesity criteria [5, 9]. Therefore, to compare prevalences among different races or ethnic groups, it is important to have the same definition of metabolic syndrome and similar study protocols in selecting population and measuring metabolic components.

In spite of the many reports on the prevalence of metabolic syndrome among Asians, direct comparison of prevalences between Koreans, Japanese, and Chinese, measured under the same study protocol, has never been done. Also, prevalence in Korean emigrants (KEs) to Japan and China has never been estimated, even though it could provide valuable information on the relative importance of ethnicity (or genetic background) and life-style change on the development of metabolic syndrome. The object of this study was to compare directly the prevalence of metabolic syndrome between KEs and their host country residents in Japan and China in the Korean Emigrant Study (KES).

## MATERIALS AND METHODS

### Subjects

The KES is a cohort study initiated to elucidate the effect of environmental change, genetic susceptibility, and their interaction on hypertension, diabetes, metabolic syndrome, and other health-related outcomes by comparing KEs and their host country residents, and Koreans living in Korea. We chose three regions near Korea, where large numbers of Koreans had migrated from 100 yr ago; Kobe-Osaka area in Japan, and Changchun and Yanbian area in Northeastern China. Changchun and Yanbian were different in that Changchun had KEs mostly migrated from the southern part of South Korea while Yanbian had KEs mostly migrated from the northern most part of North Korea. We recruited KEs who had emigrated at least 15 yr ago, and their host country residents (Japanese in Japan and Han-Chinese in China) with the ratio of 1:1. In Japan, the study was advertised by fliers, on-site visits, and introductory conferences in various Korean-Japanese societies, gatherings, companies, and schools (to recruit the parents) as well as Korean consulates in Kobe and Osaka. Participants were recruited through individual applications or group applications. Individuals were examined in Kobe Asahi hospital. For groups with 5 or more participants who were unable to visit this hospital, our research personnel (nurses and clinical technicians) went to their site to conduct the health examination. Participants took the questionnaires home from the examination sites, completed them and then mailed them to us. In China, minority races had their own villages, and KEs and Han-Chinese were recruited from their own villages. Our research personnel made home visits for the recruitment and questionnaire survey, and then conducted the health examination during a specific period of time in a school building or regional health office in each village. From May 2005 to February 2008, a total of 3,263 men and women, aged 30 or over participated in our study, and after excluding 330 participants who did not provide a complete set of data, a total of 2,933 were included in the analysis; 965 from Kobe-Osaka in Japan, 1,019 from Yanbian in China, and 949 from Changchun in China. An annual follow-up questionnaire survey and biennial follow-up health examinations are on-going to measure the outcomes.

### Life-style measurement

Participants responded to either a self-administered (in Japan) or an interviewer-administered (in China) questionnaire on life-style and diet. The life-style questionnaire, which was uniformly used in all three regions, was originally developed in Korea and then translated into Chinese and Japanese. The validity of the translation was confirmed through back-translation to Korean by other bilingual researchers than the original translators. Past medical history, family history, marital status, educational attainment, smoking, alcohol, and physical exercise were measured. Dietary intake was assessed by food frequency questionnaires which were developed in each region separately.

### Anthropometric and other clinical measurements

Well-trained examiners measured the anthropometric indices of participants with light clothing and without shoes. Height was measured to the nearest 0.1 cm and weight was measured to the nearest 0.1 kg using electronic body composition measuring devices (Yamato DF830WM in Kobe-Osaka, TANITA BC-600-WH in Yanbian and Changchun). Body mass index (BMI) was calculated by dividing weight (kg) by height squared (m^2^). Waist circumference was measured to the nearest 0.1 cm at the middle point between the lowest point of the rib cage and the iliac crest.

Blood pressure was measured twice with a 5 min interval in each arm using an automated blood pressure measurement device (TERUMO ES-H55 in Kobe-Osaka) or mercury sphygmomanometer (in Yanbian and Changchun), with participants in a sitting position. The average of the two measurements was used for analysis. Blood was drawn after at least 4-hr (in Japan) to 8-hr (in China) fasting and urine was also collected. Blood chemistries were conducted in clinical labs affiliated to this study in each region. Fasting glucose, triglycerides, and HDL-cholesterol were measured by autoanalyzer (Hitachi 7170 autoanalyzer in Kobe-Osaka, Hitachi 7600 in Yanbian, and Beckman Synchron LX20 in Changchun). Serum and plasma were separated from the whole blood within 1 hr of blood collection, and DNAs were extracted from the whole blood and stored at -80℃ in each region and then transferred to Korea.

### Definition of metabolic syndrome

The definition of metabolic syndrome in this study was NCEP-ATP III with Asian-Pacific criteria for abdominal obesity (waist circumference >90 cm for men and >80 cm for women) for easier comparison with results from other Korean studies. According to NCEP-ATP III, a person was diagnosed as having metabolic syndrome if three of following criteria were satisfied; abdominal obesity, high fasting triglyceride (>=150 mg/dL), low HDL-cholesterol (<40 mg/dL for men, <50 mg/dL for women), high blood pressure (systolic >=130 mmHg or diastolic >=85 mmHg or current medication for hypertension), and high fasting glucose (>=110 mg/dL or current medication for diabetes). Other definitions, such as revised NCEP-ATP III, WHO and IDF criteria, were also applied for the easy comparison with other studies and to see if the differences in prevalences hold true under other definitions.

### Statistical analysis

We used the χ^2^ test and Mantel-Haenszel χ^2^ test stratified by age group to compare demographic characteristics of KEs and host country residents. Age adjusted prevalence of metabolic syndrome was calculated by the direct standardization method using the 2005 Korean Census population as a standard population. Prevalences of metabolic syndrome and prevalences of metabolic components were compared between KEs and their host country residents using logistic regression adjusted for age. We examined the relative importance of the components of metabolic syndrome in diagnosing metabolic syndrome for each country and sex group using the standardized coefficients from logistic regression. The standardized coefficient of a component of metabolic syndrome can be interpreted as the average increase in log-odds of metabolic syndrome in standard deviation scale by one standard deviation increase of the component. Standardized coefficients make it possible to assess the relative importance of different components of metabolic syndrome, even when they are measured in different units [10]. We used the SAS system version 9.1 for all the analyses.

This study was approved by Samsung Medical Center Internal Review Board, Hanyang University Hospital Internal Review Board, the Ethics Committee in Kobe Asahi Hospital and the Ethics Committee in Yanbian University. Written informed consent was obtained from each participant before inclusion in this study.

## RESULTS

For the general characteristics of the participants, age was significantly different between ethnic groups in men in Kobe-Osaka, men and women in Yanbian, and women in Changchun (Table 1). Age was controlled for in all the rest of the analyses. KEs were less educated than Japanese in Kobe-Osaka. There were no significant differences in educational attainment between KEs and Chinese in both regions in China. Current smoking rate and alcohol drinking rate varied by region and sex and inter-ethnic differences were different by regions. There was no significant difference in exercise rate between KEs and host country residents in all regions.

**Figure 1:**
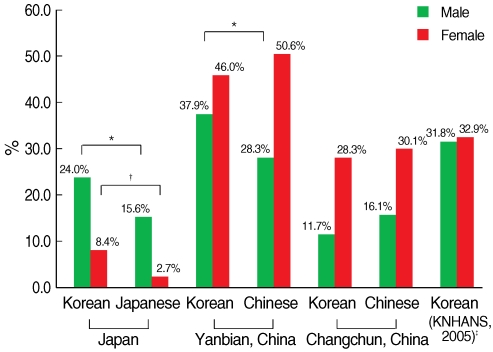
Age-adjusted^§^ prevalence of metabolic syndrome according to the NCEP-ATP III with Asian-Pacific abdominal obesity criteria. ^*^p<0.05, ^†^p<0.01; Comparisons of prevalences of metabolic syndrome between Korean emigrants and host country residents were conducted within the same country and sex group. p-values were calculated using logistic regression adjusted for age. ^‡^Prevalences in Korea were taken from the result of The Third (2005) Korea National Health and Nutrition Examination Survey (KNHANS III) using revised NCEP-ATP III definition (Ministry of Health, Welfare and Family Affairs, 2006); ^§^Age-adjusted prevalence was calculated using the direct standardization method based on the 2005 Korean census population obtained from the Korea National Statistical Office.

The KEs in Kobe-Osaka had significantly higher age-adjusted prevalences of metabolic syndrome than Japanese in both men and women (in men 24.0% vs. 15.6%, p=0.04, in women 8.4% vs. 2.7%, p=0.01), while KEs in Changchun had similar age-adjusted prevalences to Chinese (in men 11.7% vs. 16.1%, p=0.37, in women 28.3% vs. 30.1%, p=0.91) (Figure 1). The age-adjusted prevalences in Yanbian were generally higher than other regions, and the differences in prevalence between KEs and Chinese were significant among men but not among women (in men 37.9% vs. 28.3%, p=0.03, women 46.0% vs. 50.6%, p=0.44). Men had higher age-adjusted prevalence than women in Japan (19.5% in men and 6.0% in women, p<0.001), but men had lower age-adjusted prevalence than women in Yanbian (33.3% in men and 47.9% in women, p<0.001) and Changchun (13.3% in men and 28.6% in women, p<0.001) (data not shown in table).

When men and women were taken together, KEs in Kobe-Osaka had a significantly higher age-adjusted prevalence of metabolic syndrome than Japanese, but KEs in Yanbian and Changchun had a similar age-adjusted prevalence to Chinese (Table 2). The age-adjusted prevalences of metabolic syndrome varied according to the definition of metabolic syndrome. The difference in prevalence between KEs and Japanese existed only under the NCEP-ATP III and revised NCEP-ATP III definition.

Among the components of metabolic syndrome, high blood pressure showed the highest age-adjusted prevalence in all ethnic-sex specific groups in Kobe-Osaka (Table 3). High blood pressure and abdominal obesity showed a significant difference between ethnic groups in Kobe-Osaka. Low HDL-cholesterol showed the highest age-adjusted prevalence in all ethnic-sex specific groups in Yanbian, although it was high triglyceride and abdominal obesity that showed significant differences in age-adjusted prevalence between ethnic groups. High blood pressure in men and abdominal obesity in women showed the highest prevalence in Changchun, and high blood pressure and high triglyceride in men and high triglyceride in women showed significant differences in age-adjusted prevalence between ethnic groups.

According to the standardized coefficient estimates of each component of metabolic syndrome in men, abdominal obesity had the largest effect on the prevalence of metabolic syndrome in all ethnic groups except Koreans in Japan, where high fasting glucose had the largest effect (Table 4). In women, high triglyceride was the most influential component in diagnosing metabolic syndrome in most regions, while low HDL-cholesterol had the largest effect in Japanese (Table 5).

## DISCUSSION

We observed a significantly higher age-adjusted prevalence of metabolic syndrome in KEs than in host country residents in Japan, but not in China. Also, the age-adjusted prevalence was significantly higher in men than in women in Japan, but significantly higher in women than in men in China. The components which showed significant difference in prevalence between ethnic groups were high blood pressure and abdominal obesity in Japan, and mostly triglyceride in China. The most influential component in diagnosing metabolic syndrome was abdominal obesity in men and triglyceride in women.

The prevalences we observed in this study were fairly comparable to other studies previously conducted in Korea, Japan, and China. The Korean national prevalences of metabolic syndrome in the Third (2005) National Health and Nutrition Examination Survey were 31.8% for men and 32.9% for women by the revised NCEP-ATP III definition [11], prevalence in Japan was 7.8% by the definition of the Japanese Committee for the Diagnostic Criteria of Metabolic Syndrome (JCD-CMS) in 2005 according to the data from The Research Group on Serum Lipid Level Survey 2000 in Japan [6], and prevalence in rural China was 4.9% by the ATP-III definition according to the data from a rural population of 18,630 in Anhui province [12]. In our study, the age-adjusted prevalences of metabolic syndrome in KEs varied by region; 18.7% in Kobe-Osaka, 44.0% in Yanbian, and 25.5% in Changchun (Table 2). KEs living in Yanbian had the highest prevalence, probably due to the high prevalence of abnormal HDL-cholesterol level. This unusually high prevalence of abnormal HDL was re-checked by our colleagues in Yanbian but the result was the same. The prevalence of metabolic syndrome among Japanese in Arai et al. [6] study was lower than our study (14.5%), partly due to the stricter criteria for abdominal obesity for women by the JCDCMS (waist circumference >=85 cm for men and >=90 cm for women) than by the NCEP-APT III. The prevalence of metabolic syndrome in Chinese varied in our study (44.8% in Yanbian and 25.8% in Changchun), and so did the reported prevalences in previous studies conducted in various regions in China. This variation in prevalence among Chinese populations may be understandable considering the size of the country and differences in study methods. One of the studies surveyed 447 persons in the same area as our study, Yanbian, and reported the prevalences as 36.2% in men and 44.8% in women among Korean-Chinese (KEs in our study), and 7.3% in men and 12.2% in women among Han-Chinese [13]. The prevalences among Korean-Chinese were similar to our study while those among Han-Chinese were much lower than ours. The prevalences of metabolic syndrome among emigrants to other countries where diet and life-style are markedly different from their home country tend to follow the prevalences of the host country [14]. However, we did not observe such a trend among KEs living in Japan.

The most discriminating components between ethnic groups were high blood pressure and abdominal obesity in Japan, and mostly triglyceride in China. Since the smoking rate is lower in KEs than Japanese and the current exercise rate is not different between ethnic groups, these differences in high blood pressure and abdominal obesity rates cannot be easily explained by the differences in lifestyle. More detailed analysis on exercise, diet, disease history might give us possible risk factors.

According to the standardized coefficient analysis, the most influential component in diagnosing metabolic syndrome was abdominal obesity in men and triglyceride in women. Kanjilal et al. [15] presented the percent contribution of individual components of metabolic syndrome, a different method to show the relative importance of a metabolic component. Their study population was Indians from Bangalore and Mumbai. The greatest contributing factor among all the metabolic components was waist to hip ratio for men and HDL-cholesterol/waist circumference/BMI for women. Kim et al. [3] reported odds ratios of cardiovascular diseases for each component as a dichotomous variable defined by NCEP-ATP III criteria using KNHANES data collected in 1998. They showed that low HDL-cholesterol in men and hypertriglyceridemia in women showed the biggest odds ratio. In both Kanjilal et al.'s study and our study, abdominal obesity was one of the most influential components in metabolic syndrome. However, the relative importance of a metabolic component may vary depending on the characteristics of the population in the study and the method of analysis.

There are some limitations in our study. Although we tried to standardize our surveys in the three different regions as uniformly as possible, we had to modify some procedures according to the local research environment. The method of population recruitment and clinical measurements had to be decided in ways that would best accommodate the local situation. For example, we conducted health examinations in Japan in both mornings and afternoons in order to accommodate many small-group on-site examinations, whereas a large examination conducted in the morning during a specific period was possible in China. As a result, the minimum fasting time was 4 hr in Japan, which was decidedly shorter than the 8 hr of fasting time in China. This may have made us overestimate the prevalence of metabolic syndrome in Japan, although the comparison of prevalences between ethnic groups within Japan may not have been affected. Also, the prevalences in Japan were still lower than the other regions. Because of the slight differences in survey procedures, we did not statistically compare the prevalences between regions. In addition, the prevalence in our study might lack some representativeness because the participants in each region were not selected randomly and the number of participants in each region was only moderate.

However, this study is the first report that measures the prevalences of metabolic syndrome in KEs in Japan and China, and compares them directly with the prevalences in their respective host country residents in multiple regions of Asia. The method of recruiting participants and measuring metabolic components may have been slightly different region by region, but they were uniform at least within the region. Also, all three regions were surveyed in the same period of time with the same life-style questionnaires and health examination protocols. Therefore, the comparison of prevalences between ethnic groups within each region may not have been greatly biased.

We observed a significant difference in prevalence of metabolic syndrome between KEs and Japanese but no such difference between KEs and Chinese. In Kobe-Osaka, KEs tended to be less educated than Japanese and KE women tended to smoke less than Japanese women, but these features do not give us a full understanding of the reasons for such a difference in prevalence. Further study is needed to elucidate the reasons for the differences in prevalence of metabolic syndrome between KEs and Japanese and the similarities between KEs and Chinese, by comparing detailed life-style characteristics (including diet) and genetic make-up among the three ethnic groups.

## Figures and Tables

**Table 1:**
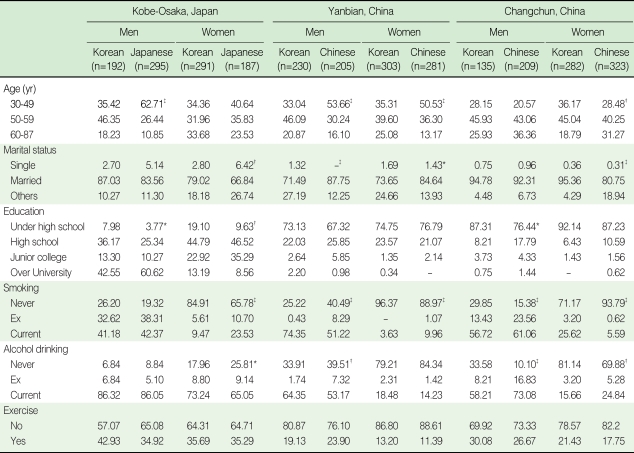
Demographic characteristics of the participants recruited between 2005 and 2007 in Kobe-Osaka, Japan, Yanbian, China, and Changchun, China in Korean Emigrant Study (unit: %)

^*^p<0.05; ^†^p<0.01; ^‡^p<0.001; p-values were calculated using the Mantel-Haenszel χ^2^ test comparing age-adjusted distributions of factors between Korean emigrants and host country residents within the same country and sex group.

**Table 2:**
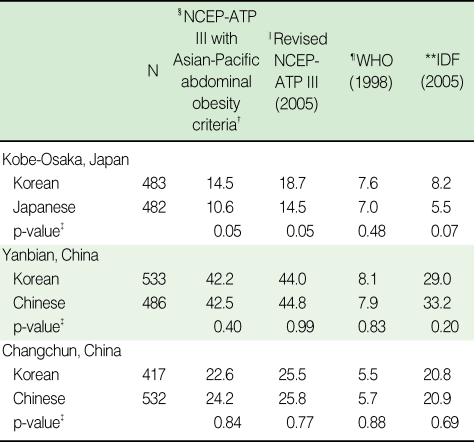
Age-adjusted prevalence^*^ of metabolic syndrome by ethnic groups in Kobe-Osaka, Yanbian, and Changchun according to different definitions of metabolic syndrome (unit: %)

^*^Age-adjusted prevalence was calculated using the direct standardization method based on the 2005 Korean census population obtained from the Korea National Statistical Office; ^†^These criteria were used primarily in this paper; ^‡^Comparison of prevalences of metabolic syndrome between Korean emigrants and host country residents were conducted within the same country. p-values were calculated using logistic regression adjusted for age; ^§^Three or more of the following: waist circumference >90 cm (M) or >80 (F), blood pressure >=130/85 mmHg, HDL-cholesterol <40 mg/dL (M) or <50 mg/dL (F), triglyceride >=150 mg/dL, fasting glucose >=110 mg/dL; ^∥^Three or more of the following: waist circumference >=90 cm (M) or >=85 (F), blood pressure >=130/85 mmHg, HDL-cholesterol <40 mg/dL (M) or <50 mg/dL (F), triglyceride >=150 mg/dL, fasting glucose >=100 mg/dL; ^¶^{Waist to hip ratio >0.9 (M) or >0.85 (F)} or BMI >30 kg/m^2^ plus two or more of the following: blood pressure >=140/90 mmHg, HDL-cholesterol <35 mg/ dL (M) or <39 mg/dlL (F), triglyceride >150 mg/dL, fasting glucose >=110 mg/dL; ^**^{Waist circumference >=94 cm (M) or >=80 cm (F)} plus two or more of the following: blood pressure >=130/85 mmHg, HDL-cholesterol <40 mg/dL (M) or <50 mg/dL (F), triglyceride >=150 mg/dL, fasting glucose >=100 mg/dL.

**Table 3:**
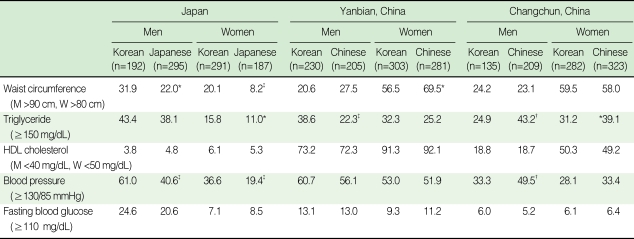
Age-adjusted prevalence^§^ of components of the metabolic syndrome according to the NCEP-ATP III with Asian-Pacific abdominal obesity criteria (unit: %)

HDL, high density lipoprotein. ^*^p<0.05, ^†^p<0.01, ^‡^p<0.001; comparison of prevalences of components of metabolic syndrome between Korean emigrants and host country residents were conducted within the same country and sex group. p-values were calculated using logistic regression adjusted for age. ^§^Prevalence is age-adjusted using the direct standardization method based on the 2005 National Census obtained from the Korea National Statistical Office.

**Table 4:**
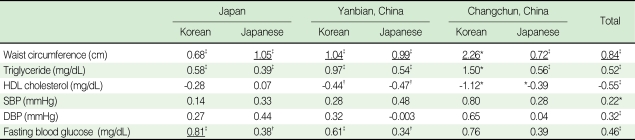
Standardized coefficient estimates of the components of metabolic syndrome among men using logistic regression

HDL, high density lipoprotein; SBP, systolic blood pressure; DBP, diastolic blood pressure. ^*^p<0.05, ^†^p<0.01, ^‡^p<0.001; p-values are for the significance of standardized coefficient estimates in the logistic regression model. __, Highest coefficient among the metabolic components within a specific ethnic group.

**Table 5:**
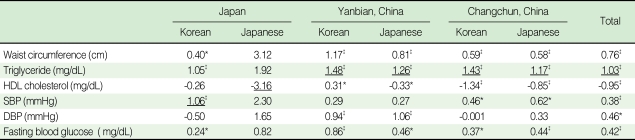
Standardized coefficient estimates of the components of metabolic syndrome among women using logistic regression

HDL, high density lipoprotein; SBP, systolic bloodp pressure; DBP, diastolic blood pressure. ^*^p<0.05, ^†^p<0.01, ^‡^p<0.001; p-values are for the significance of standardized coefficient estimates in the logistic regression model. __, Highest coefficient among the metabolic components within a specific ethnic group.
